# Microbial communities on station and train surfaces in Chennai Metro: insights into urban transit microbiome

**DOI:** 10.1128/msphere.00433-26

**Published:** 2026-10-08

**Authors:** Vijaya Yuvaram Singh, Veerendra P. Gadekar, Srijith Sasikumar, R. M. Rajeeva Lokshanan, V. Senthamizhan, Bharath Prithiviraj, Himanshu Sinha, Karthik Raman

**Affiliations:** 1 Department of Biotechnology, Bhupat and Jyoti Mehta School of Biosciences, IIT Madras 37268, Chennai, India; 2 Centre for Integrative Biology and Systems Medicine, Wadhwani School of Data Science and Artificial Intelligence, IIT Madras 37268, Chennai, India; 3 Wadhwani School of Data Science and Artificial Intelligence, IIT Madras 37268, Chennai, India; 4 Department of Data Science and Artificial Intelligence, Wadhwani School of Data Science and Artificial Intelligence, IIT Madras 37268, Chennai, India; 5 Reckitt Microbiome & Microbiology R&D Science Platforms, Montvale, New Jersey, USA; Tokai Daigaku Igakubu Daigakuin Igaku Kenkyuka, Isehara, Kanagawa, Japan

**Keywords:** metagenomics, whole-genome sequencing, urban microbiome, urban transit systems, surface-associated microbial communities, pangenome analysis, antimicrobial resistance (AMR)

## Abstract

Urban public transport systems, particularly metro networks, serve as key hubs for microbial transmission, yet the urban microbiome in densely populated regions like India remains poorly characterized. Understanding these environments is crucial for public health, especially in light of the COVID-19 pandemic and growing concerns about antimicrobial resistance (AMR). This study is the first of its kind to investigate the microbial communities and the presence of AMR genes in the Chennai Metro system. We collected 96 surface swabs from 12 metro stations across two lines, focusing on surfaces that people frequently touch, such as handrails, kiosks, banisters, and ticket counters. Of the collected samples, 47 met quality control standards and were subjected to shotgun metagenomic sequencing, and 41 samples with more than 1 million reads were included in our analysis. Our findings indicate that surface type significantly influences microbial community structure, with kiosks exhibiting the highest microbial diversity. Comparative analysis with global urban data sets revealed unique microbial patterns specific to Chennai, including nine species that were notably more prevalent in our samples than in other urban transit systems worldwide. Furthermore, through pangenome analysis, we generated high-quality metagenome-assembled genomes that elucidated the adaptive strategies of dominant microbial species in this urban environment. Despite their relatively low abundance, several AMR families were widely distributed across the data set, with over 80% of samples containing at least one associated AMR gene, including families linked to rifamycins, multidrug resistance, and sulfonamides. This study lays a foundation for understanding the urban microbiome in India, emphasizing distinct regional characteristics and underscoring the need for sustained monitoring to mitigate disease transmission risks in high-density transit settings.

**IMPORTANCE:**

Densely populated urban transit systems are critical hubs for microbial exchange, yet the mass transit microbiomes of Indian cities remain largely uncharacterized, representing a significant gap in global surveillance. Our study provides the first comprehensive metagenomic analysis of the Chennai Metro, a high-traffic transit network serving millions. Our findings highlight the unique environmental drivers shaping urban microbiota in India. Chennai, with over 12 million residents and a metro system serving over 105 million passengers annually, is an ideal yet uncharacterized environment for studying microbial dynamics, surface–microbe interactions, and environmental antimicrobial resistance reservoirs.

## INTRODUCTION

Public transport systems represent a dynamic environment for microbial transmission, facilitating daily interactions among diverse populations and their microbiomes. The metro system, in particular, acts as a hub for a wide range of microorganisms, including beneficial commensals and symbionts, as well as potentially pathogenic bacteria, making it a potential vector for the spread of infectious diseases (1, 2). As critical infrastructure in urban settings, public transportation systems significantly influence the microbial ecology of cities. Global initiatives, such as the MetaSUB consortium (3, 4), have advanced our understanding of urban microbiomes by characterizing the unique microbial profiles of cities around the world, providing crucial data for urban planning and public health interventions. Despite the increasing number of studies on urban microbiomes, densely populated Indian cities remain largely unexplored. As one of the fastest-growing urban centers in the world, Chennai, with a population exceeding 12 million (5), exemplifies the dramatic transformation of Indian cities in the 21st century. This study represents the first comprehensive analysis of urban microbiomes in public transportation systems in India, offering critical insights into the unseen biological ecosystems that thrive in these densely populated spaces.

Understanding the microbial communities in public transit systems is crucial for urban public health and global antimicrobial resistance (AMR) surveillance. AMR, often described as a hidden pandemic (6), has intensified due to rising antibiotic use during health crises such as the COVID-19 pandemic. In a metropolis like Chennai, where environmental factors, human activity, and rapid urbanization intersect, the dynamics of microbial communities, including their potential to harbor AMR genes, are still poorly understood. Unlike subway systems in extensively studied cities, Chennai’s unique socio-environmental landscape presents a distinct microbial signature, making it a key region for global comparative microbiome studies.

Recent advancements in next-generation sequencing (NGS) technologies have revolutionized our understanding of microbial communities, particularly those that are difficult to cultivate in the laboratory. The increasing availability of NGS data in public repositories has facilitated unprecedented opportunities to track the dissemination of microorganisms within urban environments (7–9). Investigating bacterial communities in regions severely affected by the COVID-19 pandemic is crucial for understanding the dynamics of AMR gene dissemination, which may have been amplified by the increased use of antibiotics during the pandemic (10, 11).

Urban microbiome research has experienced significant growth in recent years, driven by initiatives such as the MetaSUB consortium, which aims to map microbial ecosystems in cities worldwide. Yet, despite its vast and densely populated urban centers, India remains largely unexplored in this context. This study addresses that gap by characterizing the Chennai Metro’s microbiome, uncovering distinct microbial signatures, including nine core species that have not been previously identified as core species in other global cities. These findings underscore the need to examine how urban environments in developing nations shape microbial diversity and AMR patterns.

By characterizing the microbial landscape of Chennai’s Metro system, we provide the first window into how such environments in India may serve as reservoirs for both beneficial and pathogenic microorganisms. Moreover, this work is a call to action to implement continuous surveillance systems in urban transit environments, where rapid microbial transmission could pose heightened risks during future public health crises.

## MATERIALS AND METHODS

### Sampling microbial communities in the Chennai Metro stations

**Fig 1:**
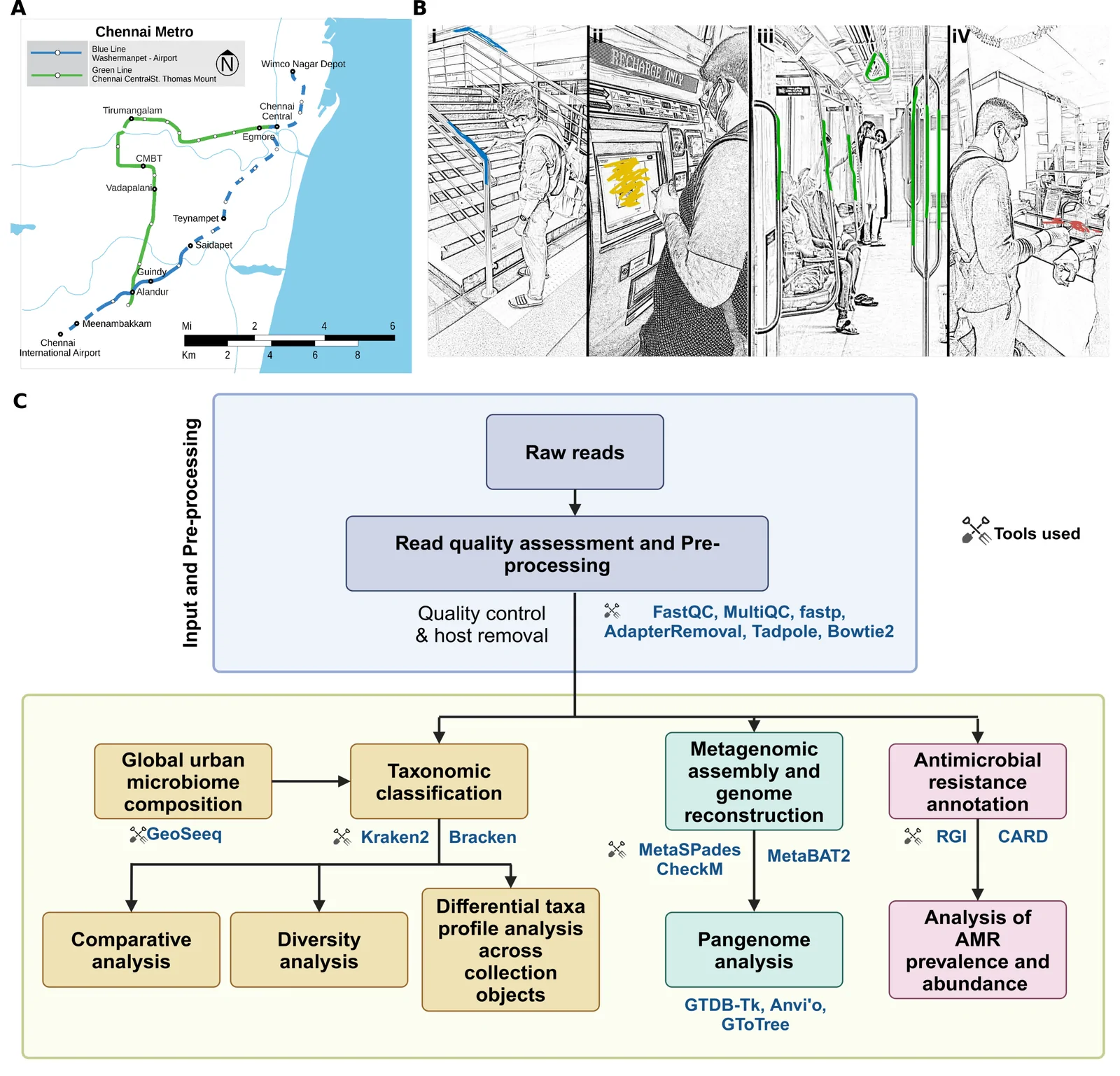
(**A**) Chennai Metro line map highlighting sample collection stations (12). (**B**) Samples were collected from various surfaces, including (i) handrails in metro stations (banisters), (ii) ticket vending machines (kiosks), (iii) handrails in trains (rods), and (iv) ticket counters. The swabbing areas on these surfaces are highlighted in blue, yellow, green, and red, respectively. To maintain consistency across all samples, only a portion of each surface was swabbed rather than the entire surface. (**C**) Workflow for data processing and analysis.

To explore microbial communities within the Chennai Metro system, we collected samples (*n* = 96) from 12 metro trains and stations along the Blue and Green Lines, operated by Chennai Metro Rail Limited (CMRL; Fig. 1A). The Blue Line spans 32.65 km, connecting Wimco Nagar Depot to Chennai International Airport, while the Green Line covers 22 km from Chennai Central to St. Thomas. Sampling occurred in two batches: December 2021 and January 2022. Only the samples passing the quality control (QC) criteria were subjected to shotgun metagenomic sequencing (Method S1). A detailed list of the samples that passed the QC and were used for analysis is provided in Table S1. The DNA extraction, library preparation, and sequencing were outsourced, and the details of the QC, sequencing, raw read processing, and mapping can be referred to in Methods S2 and S3.

### Taxonomic profiling and comparative microbiome analysis

Taxonomic classification and species-level abundance estimation were performed using Kraken2 v2.1.3 and Bracken v2.7 within the CAMP pipeline (13). Species prevalence across samples was used to define core and subcore taxa. For global comparisons, MetaSUB urban microbiome data sets were downloaded and filtered to retain only surface samples. All external data sets were processed using the same pipeline to ensure consistency. Species were categorized into core, subcore, peripheral, and unique-core groups and compared across cities. We also performed a bootstrap analysis to control for variations in the number of samples per city and sample collection objects. Detailed workflows and filtering criteria are provided in Methods S4 and S5.

### Microbial diversity and differential abundance analyses

Microbial diversity analyses were conducted in R using phyloseq v1.44.0 (14). Alpha diversity was measured using the Shannon index, and beta diversity was assessed using Bray–Curtis dissimilarity and principal coordinate analysis (PCoA). Statistical significance was evaluated using Wilcoxon, Kruskal–Wallis, and PERMANOVA tests with BH correction. Differential abundance across surface types was assessed using MaAsLin2 v1.15.1 (15) with a one-versus-rest design. Taxa with an absolute log fold change |log_2_FC| >1 and *P*_adj_ <0.05 were considered significant. Expanded statistical and modeling details are provided in Methods S6 and S7.

### Genome reconstruction and pangenome analysis

Metagenomic reads were assembled using MetaSPAdes v3.15.5 (16) and binned into metagenome-assembled genomes (MAGs) using MetaBAT2 v2.2.7 (17). Genome quality was assessed with CheckM v1.0.1 (18). High-quality MAGs were taxonomically annotated using GTDB-Tk v2.1.1 (19) and analyzed in Anvi’o v8 (20) for pangenome characterization. Phylogenomic relationships were inferred using GToTree v1.8.4 (21). Detailed assembly, binning, and pangenome parameters are described in Methods S8 and S9.

### AMR gene profiling

AMR genes were identified using RGI with CARD (22) v3.2.6. A threshold of >80% coverage of the reference sequence is used as the filtering criterion to consider an AMR gene. Abundance was normalized to reads per kilobase of transcript per million mapped reads (RPKM). AMR classes were interpreted using the WHO Access, Watch, Reserve (AWaRe) framework (23). Complete AMR analysis parameters are provided in Method S10.

## RESULTS

### Taxonomic classification and comparative analysis of distinct microbial signatures

**Fig 2:**
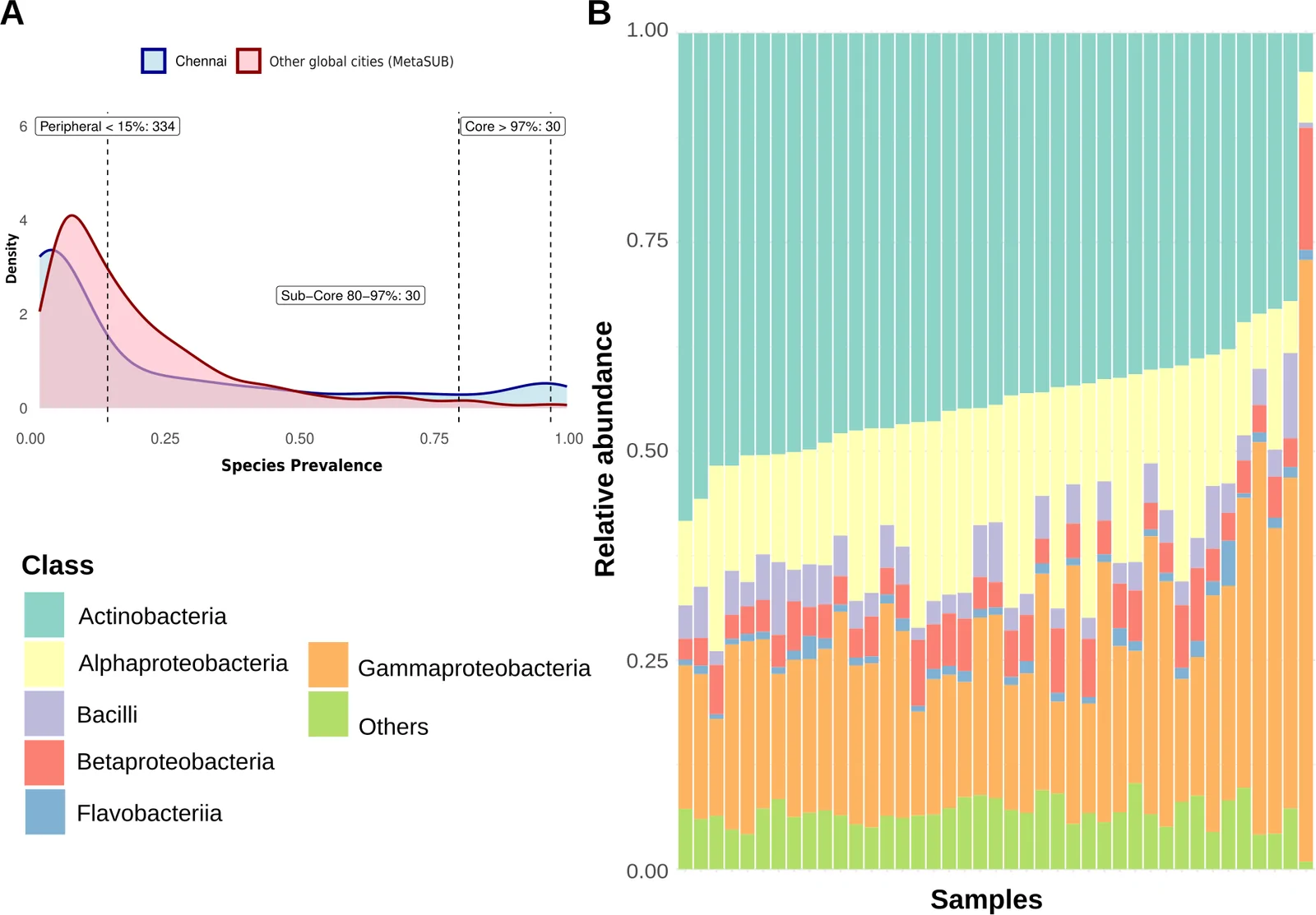
Species prevalence and community composition in the Chennai Metro. (**A**) The distribution of species prevalence indicates the proportion of samples where a species was found at a relative abundance greater than 0.001. This is illustrated in the 41 samples from Chennai (blue) compared to 2,461 surface samples from 31 other MetaSUB cities (red). Dashed lines denote the prevalence thresholds used to categorize taxa as peripheral (less than 15% of samples, *n* = 334), subcore (80%–97%, *n* = 30), and core (greater than 97%, *n* = 30). (**B**) Relative abundance of bacterial classes across 41 Chennai samples.

We first investigated how microbial species are distributed across the environments of Chennai Metro stations (Fig. 1). Specifically, we aimed to determine whether the urban environment exhibited a homogeneous microbial ecosystem or comprised distinct yet interconnected communities, particularly with respect to biodiversity. To achieve this, we examined the prevalence of species, defined as the fraction of samples in which a given taxon was found at different thresholds of relative abundance. We identified a right-skewed distribution characterized by a prominent primary peak on the left, representing the majority of species with low prevalence. A smaller secondary peak on the right highlighted a subset of species with distinct characteristics or higher prevalence within the sampled ecosystem (Fig. 2A). This allowed us to categorize the taxa into three distinct groups: “core” taxa, present in >97% of samples, “subcore” taxa, present in 80%–97% of samples, and “peripheral” species, observed in fewer than 15% of samples. Table S2 summarizes the number of identified species in Chennai samples across these distinct groups based on their relative abundance.

At a relative abundance of 0.001, which corresponded to more than 1,100 reads supporting a given species (based on the minimum library size shown in Table S3), we identified 30 core species, 30 subcore species, and 334 peripheral species in the Chennai samples (File S2, sheet 1). Most samples were dominated by species from three classes: *Actinobacteria*, *Alphaproteobacteria*, and *Gammaproteobacteria*, although the relative abundance of these classes varied (Fig. 2B).

Geographical regions and urban environments significantly influence the composition and diversity of microbial communities, shaped by factors such as climate, air quality, human activities, and the built environment (4, 24). To explore these effects, we compared samples from Chennai with those from 31 other global cities (Table S4), aiming to identify common and unique patterns in species distribution. We identified microbial signatures unique to Chennai. To emphasize these, we created a category called “unique core,” which included core species specific to Chennai that were not core or subcore in any other city at the same relative abundance. We found nine such species that were part of the core in Chennai but not in other global cities (Table 1; Table S2). To assess the robustness of the Chennai-specific unique-core species, we performed a resampling-based analysis controlling for differences in sample size across cities. None of the trials showed a statistically significant overlap with the Chennai unique-core species (File S1; Fig. S1). To further account for variation in sampling surface types, we restricted the MetaSUB data set to surface categories comparable to those sampled in Chennai, reducing the data set from 2,461 to 700 samples. Even after controlling for both sample count and surface type, no resampling trial recovered the Chennai-specific unique-core species (File S1; Fig. S2).

**TABLE 1:** Nine species differentially prevalent in Chennai city and the habitats in which they are typically found^*a*^

Species	Sources
*Brachybacterium* sp. SGAir0954	Isolated from tropical air collected in Singapore (25)
*Brachybacterium faecium*	Isolated from poultry deep litter (26)
*Brachybacterium saurashtrense*	Isolated from the roots of *Salicornia brachiata*, an extreme halophyte (27)
*Pseudomonas mendocina*	Found in water and soil samples (28)
*Pseudomonas alcaligenes*	Commonly found in soil and water (29)
*Staphylococcus arlettae*	Found in both environmental and human sources (30–32)
*Sphingobium yanoikuyae*	Found in environments ranging from terrestrial to aqueous habitats, including seawater (33)
*Comamonas aquatica*	Found in water sources (34)
*Brachybacterium* sp. P6-10-X1	Isolated from deep-sea sediments (35)

^*a*^ These species are selected based on a threshold of <80% prevalence at a relative abundance of >0.001 in other global cities, whereas they are >97% prevalent in Chennai at the same relative abundance.

Among these, *Brachybacterium saurashtrense*, which is adapted to high salinity, was first isolated from the roots of the halophytic plant *Salicornia brachiata* (27). Its presence indicates that Chennai’s coastal climate and humidity support its growth. Similarly, *Brachybacterium* sp. SGAir0954, previously isolated from tropical air in Singapore (25), thrived in Chennai’s tropical and humid conditions. *Brachybacterium faecium* (26) is typically associated with poultry environments. The presence of human-associated species, such as *Staphylococcus arlettae* (30–32), commonly found on the skin, highlighted the roles of metro passengers and urban density in microbial spread. Soil- and water-associated species, such as *Pseudomonas mendocina* (28), *Pseudomonas alcaligenes* (29), *Sphingobium yanoikuyae* (33), and *Comamonas aquatica* (34), likely reflected the local soil and water microbiome, demonstrating resilience to pollutants and potential for bioremediation in urban ecosystems. Similarly, *Brachybacterium* sp. P6-10-X1, originally isolated from deep-sea sediments (35), represents another environmental *Brachybacterium* associated with a marine sediment habitat.

### Microbial diversity and composition across various surface types

**Fig 3:**
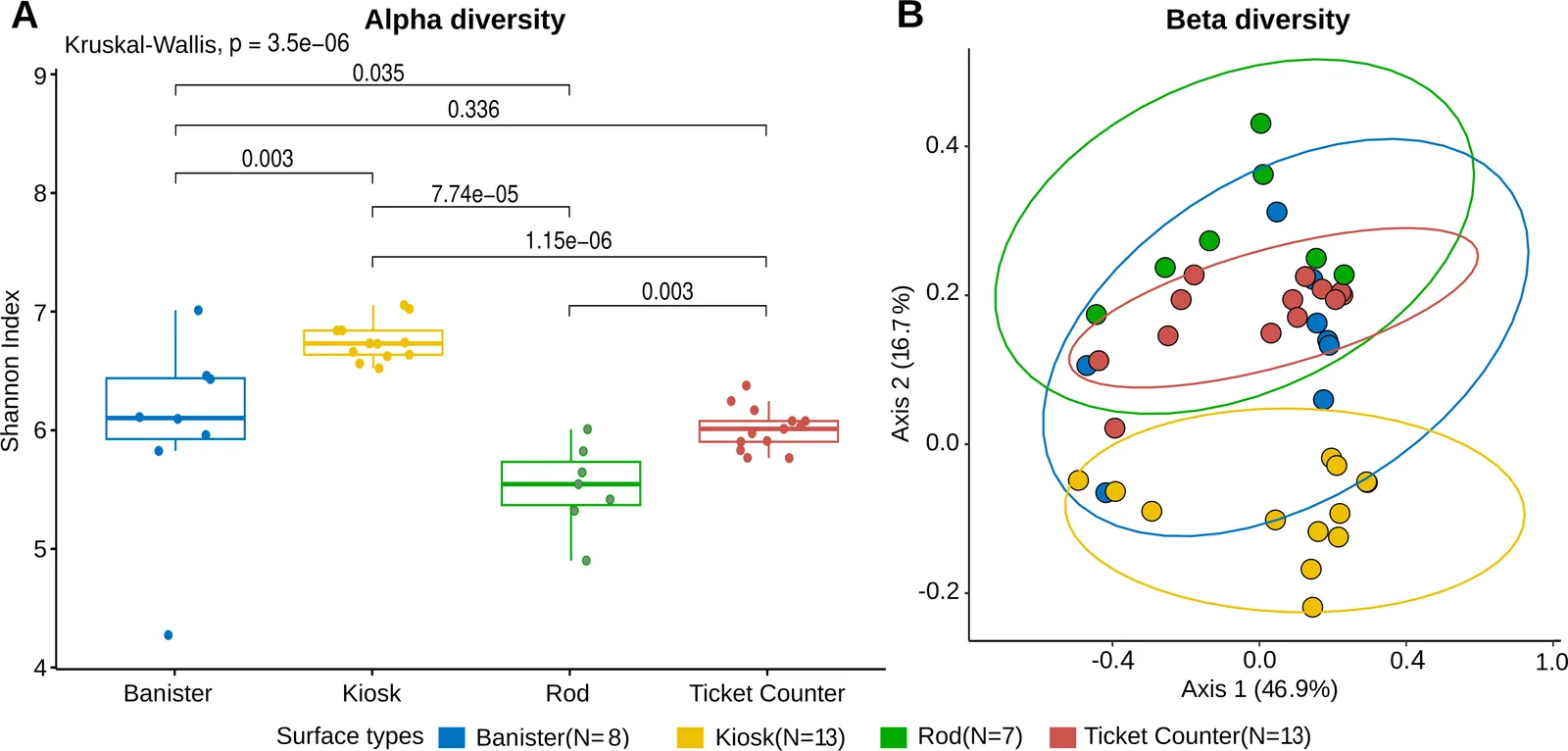
(**A**) Alpha diversity and (**B**) beta diversity of Chennai samples collected from four different surface types. A “one-versus-rest” comparison was performed for each surface type in both analyses. For alpha diversity, Wilcoxon pairwise tests were used to compute *P* values. For beta diversity, PERMANOVA was conducted to assess group-wise differences. The alpha-diversity comparisons yielded statistically significant (*P*_adj_ < 0.05) differences between kiosk and other surface types. Beta-diversity comparisons with kiosks showed significant differences for Rod (*P*_adj_ = 0.02) and Ticket Counter (*P*_adj_ = 0.04), while banister was not significantly different (*P*_adj_ = 0.17).

We evaluated the quantity and diversity of microbial communities across different surface types by analyzing alpha and beta diversity. We used the Shannon index to assess alpha diversity, which accounts for both species richness (the number of species present) and species evenness (the distribution of individuals among species). The samples collected from the kiosk exhibited a significantly higher Shannon index (median = 6.73), indicating greater species diversity than on other surfaces. In contrast, the samples from the rods showed the lowest Shannon index (median = 5.51), suggesting lower diversity (Fig. 3A). For beta diversity, we performed PCoA using the Bray–Curtis dissimilarity measure to assess differences in species composition among samples from various surfaces. This analysis considered both the presence or absence of species and their relative abundances. As expected, the kiosk samples, which showed the highest species diversity, clustered separately, indicating a distinct species composition compared to samples from other surfaces (Fig. 3B).

**Fig 4:**
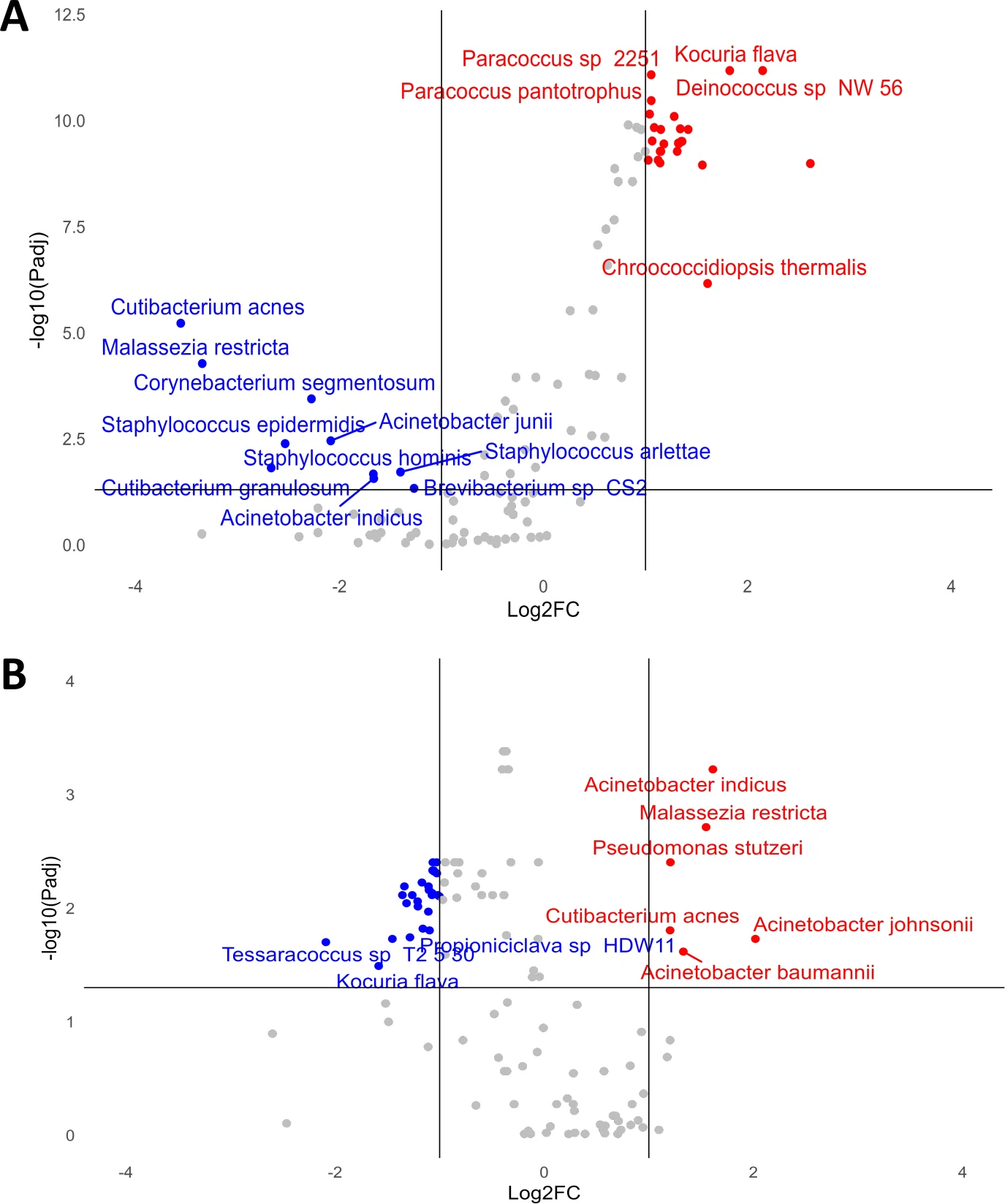
Differential representation of microbial species across surface types. (**A**) Comparison of species abundance on kiosk surfaces versus other surfaces. (**B**) Comparison of species abundance on rod surfaces versus other surfaces. Species with log_2_FC >1 or log_2_FC <−1 and *P*_adj_ <0.05 are shown in red and blue, respectively.

We further examined the variations in microbial species across different surface types. We found that 25 species were significantly enriched on kiosk surfaces (|log_2_FC| > 1 and *P*_adj_ < 0.05) compared to all other surfaces, while 6 species were significantly enriched on the rod surfaces (Fig. 4) (File S2, sheets 5 and 6). This finding aligned with our diversity analysis, in which kiosk surfaces that exhibited greater species diversity also had more species that were significantly overrepresented. In contrast, no species were significantly over- or underrepresented on the banister or ticket counter surfaces compared to the other surface types. The kiosk surfaces exhibited higher alpha diversity and a distinct microbial composition compared to other surfaces. The PERMANOVA test also showed that the kiosk samples are different from the rod (*P*_adj_ = 0.02) and the ticket counter (*P*_adj_ = 0.04). Notably, several species, such as *Kocuria flava*, *Deinococcus* sp. NW 56, *Micropruina glycogenica*, *Rhodobacter sphaeroides*, and multiple *Nocardioides* spp., were significantly overrepresented, indicating their predominance in this environment. In contrast, species like *Cutibacterium acnes*, *Corynebacterium segmentosum*, and *Staphylococcus hominis* were underrepresented in the kiosk, suggesting differential surface colonization patterns. The presence of opportunistic pathogens and resilient bacteria on high-contact kiosk surfaces underscores the importance of targeted cleaning protocols to mitigate potential health risks.

### Pangenome profiling of MAGs

**Fig 5:**
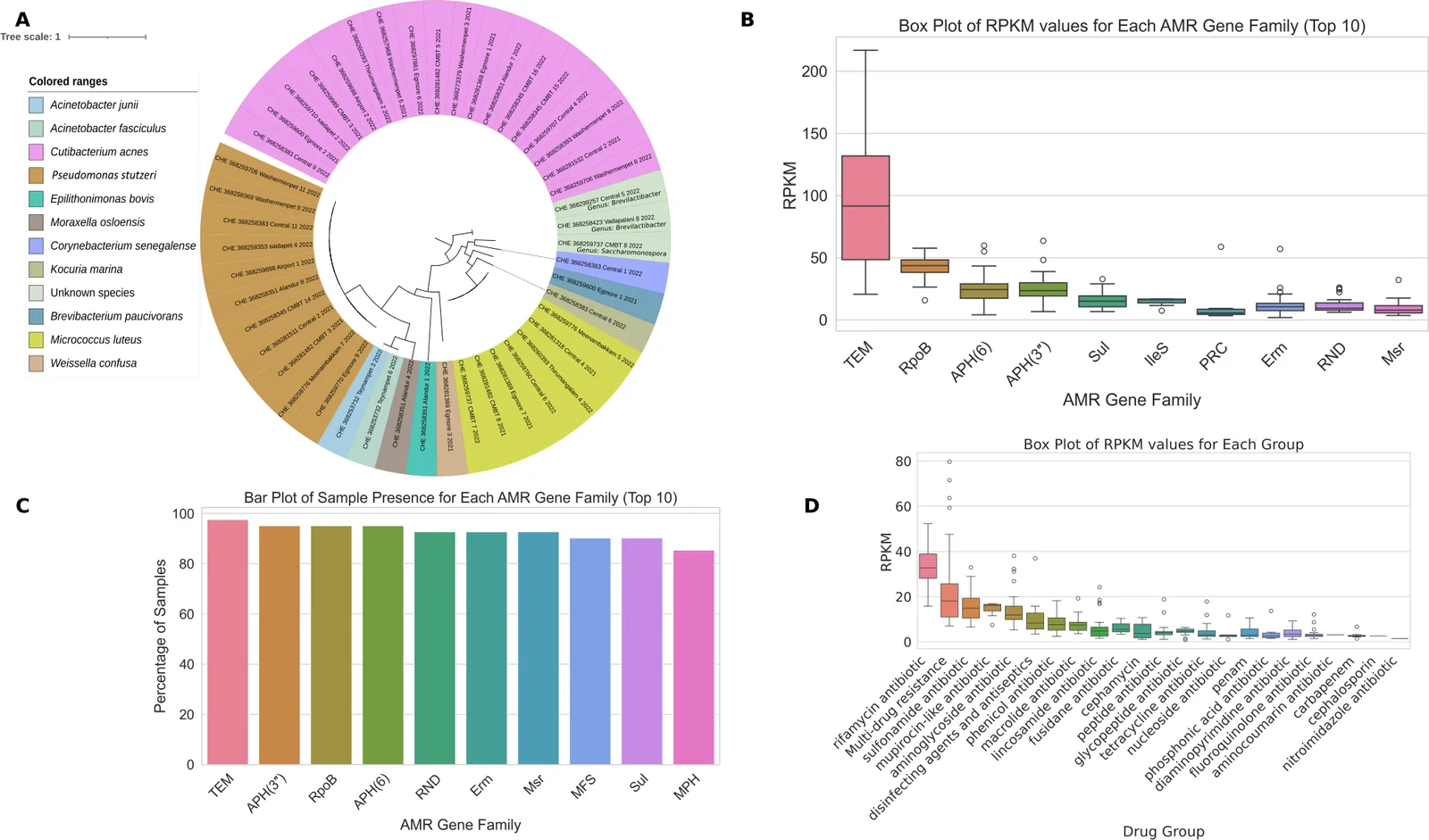
(**A**) Phylogenetic tree of the microbial species mapped to the MAGs. (**B**) Gene abundance profile represented by RPKM values for each gene family. (**C**) Frequency of sample presence (%) for the top 10 AMR gene families across the data set. (**D**) Gene abundance profiles represented by RPKM values for each drug group.

We investigated the genetic diversity and functional capabilities of microbial communities in the Chennai Metro system through pangenome analysis of MAGs. We reconstructed 47 high-quality MAGs from the samples, each satisfying the standard threshold of >90% completeness and <5% contamination, making them suitable for downstream pangenome analysis. These MAGs were initially assigned at the genus level, resulting in the identification of 12 genera (Fig. 5A). Of these, 44 MAGs spanning 10 genera could be further resolved to the species level (File S2, sheet 2). MAGs assigned to the genera *Brevilactibacter* and *Saccharomonospora* did not meet the criteria for confident species-level classification and were therefore reported as “unknown species” in Fig. 5A. We identified 11 species with average nucleotide identity (ANI) greater than 97% compared to reference genomes. The identified species included *Acinetobacter fasciculus*, *Acinetobacter junii*, *Brevibacterium paucivorans*, *Corynebacterium senegalense*, *Cutibacterium acnes*, *Epilithonimonas bovis*, *Kocuria marina*, *Micrococcus luteus*, *Moraxella osloensis*, *Pseudomonas stutzeri*, and *Weissella confusa*. Among the identified species, *Cutibacterium acnes* was represented by 18 MAGs, *Pseudomonas stutzeri* by 11 MAGs, and *Micrococcus luteus* by 7 MAGs, while the remaining species were represented by a single MAG each (Fig. 5A; File S2, sheet 2).

Our functional enrichment analysis using the COG2020 database identified a significant overrepresentation of certain functions in the MAGs from Chennai. Specifically, for *C. acnes*, we examined 18 MAGs from Chennai alongside 39 reference strains and found 10 functions that were significantly overrepresented (Table 2). In the case of *P. stutzeri*, we analyzed 11 MAGs from Chennai and 20 reference strains, revealing five functionalities that were significantly overrepresented in the Chennai MAGs (Table 3). We did not identify any significant functional enrichment in *M. luteus* MAGs.

**TABLE 2:** Significantly overrepresented functions in *Cutibacterium acnes* MAGs

COG2020 function	*P*_adj_	Log_2_FC
Ribosomal protein L36 (*RpmJ*) (PDB: 1DFE) (36)	1.5E−07	4.98
Translation initiation factor IF-1 (*InfA*) (PDB: 7PVC)	4.1E−07	4.88
Transposase *InsO* and inactivated derivatives (*Tra5*)	4.6E−07	3.98
Transposase and inactivated derivatives, IS30 family (*Tra8*)	6.7E−03	3.66
Transposase *InsE* and inactivated derivatives (*InsE*) (PDB: 2JN6) (37)	6.7E−03	3.66
Acyl-CoA thioesterase *PaaI* contains the HGG motif (*PaaI*) (PDB: 1PSU)	6.7E−03	3.66
CRISPR/Cas system-associated endoribonuclease *Cas2* (*Cas2*) (PDB: 2I0X)	1.5E−02	3.40
Multidrug transporter *EmrE* and related cation transporters (*EmrE*) (PDB: 2I68)	4.0E−02	3.07
Ca^2+^/Na^+^ antiporter (ECM27) (PDB: 3V5S)	4.0E−02	3.07
*AraC*-type DNA-binding domain and *AraC*-containing proteins (*AraC*) (PDB: 1BL0)	4.0E−02	3.07

**TABLE 3:** Significantly overrepresented functions in *Pseudomonas stutzeri* MAGs

COG2020 function	*P*_adj_	Log_2_FC
Dephospho-CoA kinase (*CoaE*) (PDB: 1JJV)	5.0E−05	4.27
Protein translocase subunit *SecG* (*SecG*) (PDB: 2AKH)	1.9E−04	4.13
Ribosomal protein S27E (RPS27A) (PDB: 1QXF)	4.2E−02	3.13
*TolB* amino-terminal domain (function unknown) (*TolBN*) (PDB: 1C5K) (38, 39)	4.2E−02	3.13
Ribosomal protein L36 (*RpmJ*) (PDB: 1DFE) (36)	2.5E−03	2.98

The enrichment of multiple transposases among the *C. acnes* MAGs indicated that these MAGs possess mobile genetic elements that could facilitate genetic diversity and adaptability. These elements could help the organism acquire new traits or resistances, particularly in changing environments (40).

### Assessment of antibiotic resistance gene abundance

We annotated 72 AMR gene families in our sample set, associated with 31 drug groups and 9 resistance mechanisms (File S2, sheet 3). Among these, the TEM beta-lactamase gene family exhibited the highest abundance based on RPKM values, whereas the majority of the remaining AMR gene families showed RPKM values below 50 (Fig. 5B). We additionally introduced a “multidrug resistance” category for genes conferring resistance to multiple antibiotic classes. Overall, the AMR gene families displayed a generally low abundance pattern across the data set (RPKM: mean = 13.2, median = 6.32) spanning different drug groups and resistance mechanisms (File S2, sheet 4). Despite their relatively low abundance, several AMR gene families were broadly distributed across the samples, with more than 80% of samples harboring at least one associated AMR gene (Fig. 5C). Notably, AMR genes associated with Rifamycins (mean RPKM = 32.2), Multidrug resistance (mean RPKM = 16.03), and Sulfonamides (mean RPKM = 15.7) exhibited comparatively higher abundance than most other antibiotic classes (Fig. 5D).

## DISCUSSION

Urban public transit systems, such as metro networks, serve as critical hubs for microbial transmission, shaping the urban microbiome through constant human activity and high population density (41). In densely populated regions like India, where millions rely on metro systems daily, these environments act as dynamic ecosystems where microbial communities interact, adapt, and spread. The Chennai Metro, operated by CMRL, has rapidly become a vital part of the city’s urban transit infrastructure. Since its launch on 29 June 2015, the metro has transported an impressive 355.3 million passengers, highlighting its critical role in urban mobility (42). In 2024 alone, CMRL recorded 105.2 million passengers, a substantial increase from 91.1 million in 2023. As metro ridership continues to grow, so does the importance of understanding its impact on microbial transmission and urban health.

This study presents the first comprehensive metagenomic analysis of an Indian transit system, revealing a unique microbial signature with nine core species that were differentially abundant in Chennai. The presence of species such as *Brachybacterium saurashtrense* and *Brachybacterium* sp. SGAir0954 suggests that Chennai’s tropical coastal environment and humidity may affect the microbial composition of the metro system.

Our environmental metagenomic findings also align with clinical evidence, highlighting region-specific challenges in antimicrobial resistance. For instance, a recent study in Vellore, Tamil Nadu, documented multidrug-resistant bacterial strains, including *Acinetobacter* species (43), in sewage receiving hospital wastewater, underscoring the selective enrichment driven by antibiotic residues and hospital effluents, which represent public health hazards through environmental dissemination. Moreover, locally prevalent resistant isolates displaying phenotypes relevant to rifamycin and sulfonamide resistance corroborate our AMR gene findings within the Chennai Metro microbiome. According to the 2019 WHO AWaRe classification framework, sulfonamides are categorized in the “access” group, whereas rifamycins belong to the “watch” group. These data reinforce the importance of including such environmental reservoirs in integrated AMR surveillance efforts.

Additionally, *Staphylococcus arlettae*, which we found highly prevalent in Chennai’s metro, is increasingly reported from clinical settings associated with medical device infections and bloodstream infections (30, 31), directly linking our environmental observations to the ecology of healthcare-associated pathogens. Opportunistic pathogens *Moraxella osloensis* and *Acinetobacter junii* are similarly known to persist in household and hospital environments within India (44), supporting their epidemiological relevance in urban microbial health risks. Therefore, monitoring these microbial communities in cities like Chennai is crucial.

The increasing risk of flooding and extreme weather events in coastal cities such as Chennai highlights the importance of continued microbial surveillance in urban transit systems (45). Environmental disturbances associated with flooding may alter microbial community composition and influence the dissemination of AMR-associated genes in public spaces.

Surface type significantly influenced microbial diversity, with kiosks exhibiting the highest alpha diversity, consistent with previous studies (24), likely due to frequent human contact. The concentrated use of kiosks as focal points for user interaction by diverse individuals can create unique microbial communities, potentially resulting in higher alpha diversity despite their localized sampling areas, which are comparatively smaller surface contact points than handrails or banisters. In contrast, the larger surface areas of handrails may support a more homogeneous microbial community due to constant exposure to human contact, as explained by ecological principles. According to these principles, continuous and extensive surface exposure favors the dominance of well-adapted taxa, thereby decreasing community heterogeneity. Meanwhile, smaller surfaces, such as kiosks, although having less total area, receive diverse and frequent contacts that vary temporally and spatially, introducing a wider array of microbial inputs. The resulting heterogeneity of kiosk microhabitats supports higher alpha diversity and greater taxonomic variability. Understanding these dynamics is crucial for developing effective cleaning protocols and public health strategies in urban environments.

We investigated the genetic diversity and functional potential of microbial communities in the Chennai Metro system by reconstructing 47 high-quality MAGs. Among these, *Cutibacterium acnes*, *Pseudomonas stutzeri*, and *Micrococcus luteus* were the most frequently represented species, suggesting their ecological dominance or adaptation to the urban transit environment. In contrast, several species were represented by a single MAG, indicating their potential niche specialization or low abundance within the ecosystem. Notably, *Moraxella osloensis*, an opportunistic pathogen commonly found in household environments, has been previously reported as a dominant species on kitchen surfaces and sponges, with its prevalence influenced by cleaning methods (44). Similarly, *Acinetobacter junii* is frequently identified in house microbiomes in India, highlighting its persistence in built environments (44). Among *C. acnes* MAGs, we observed an enrichment of transposases, suggesting the presence of mobile genetic elements that could promote genetic diversity and adaptability (40). These elements may facilitate the acquisition of new traits or resistance mechanisms, particularly under environmental stress. Additionally, the overrepresentation of the multidrug transporter *EmrE* in *C. acnes* implies a potential mechanism for exporting toxic compounds, including antibiotics, thereby enhancing its survival under antimicrobial pressures. In *P. stutzeri* MAGs, we observed an overrepresentation of dephospho-CoA kinase (*CoaE*), an enzyme critical for coenzyme A (CoA) biosynthesis (46). The significant overrepresentation of *CoaE* underscores the bacterium’s metabolic adaptability and ecological versatility in urban transit environments. CoA biosynthesis is essential for fundamental cellular processes, including energy production, fatty acid metabolism, and responses to environmental stresses such as disinfectants and oxidative agents commonly encountered on public surfaces. *P. stutzeri* is a well-documented opportunistic pathogen with the ability to persist across diverse environments, including soil, water, and clinical settings, and it has been implicated in bloodstream and respiratory infections, especially in immunocompromised individuals (47). The elevated *CoaE* activity may enhance *P. stutzeri*’s resilience and persistence on frequently touched surfaces, thereby increasing the potential for microbial transmission and colonization in crowded urban settings. Given that CoA biosynthesis enzymes also represent antimicrobial drug targets (48), this links the pathway to clinically relevant resistance mechanisms. This finding highlights a functional biomarker of microbial survival and public health risk, advocating for integrated microbial and metabolic surveillance in urban transit systems to inform cleaning and infection control strategies. Our AMR gene analysis revealed low overall abundance across most drug groups, yet their widespread presence indicates latent resistance potential. Genes associated with sulfonamide and rifamycin resistance exhibited relatively higher abundance, suggesting selective pressure. Sulfonamides act as broad-spectrum antibiotics by inhibiting dihydropteroate synthase (49), while rifamycins target bacterial RNA polymerase (50). Notably, the high prevalence of rifamycin resistance aligns with reports of its presence in hospital isolates from Chennai, indicating a possible overlap between clinical and urban microbial populations (51). This selective pressure likely arises from environmental antibiotic exposure resulting from human healthcare, agriculture, and community use, compounded by the use of disinfectants that co-select resistance traits, enabling bacterial survival in urban transit settings. Sulfonamide resistance typically involves alternative dihydropteroate synthase enzymes, while rifamycin resistance arises primarily from mutations in the RNA polymerase beta subunit, both of which reduce antibiotic efficacy.

While our bioinformatics pipelines were harmonized, variations in DNA extraction kits and sequencing platforms across global MetaSUB partners introduce inherent limitations to cross-city microbiome comparisons. Although 96 surface swabs were initially collected from the Chennai Metro system, only 41 of the 96 surface swabs passed both DNA quality control and the minimum read-depth threshold, thereby limiting statistical power. Furthermore, although the negative control samples failed DNA concentration quality checks, suggesting minimal large-scale environmental or cross-contamination, the absence of sequenced negative controls prevented the application of algorithmic background decontamination approaches. An additional limitation of DNA-based metagenomics is its inability to distinguish between viable and non-viable cells; consequently, the detection of specific taxa and antimicrobial resistance genes reflects only their environmental presence and does not directly indicate viability, transmission potential, or clinical risk. Finally, the data set represents a single temporal snapshot and does not capture seasonal or longitudinal microbial dynamics.

Our findings underscore the importance of continuous microbial surveillance in urban public transportation systems, particularly in dense and rapidly growing cities like Chennai. As urbanization accelerates and public transport networks expand, the dynamics of microbial transmission in these densely populated environments will become increasingly relevant to public health. This aligns directly with the UN Sustainable Development Goals (SDGs), particularly SDG 3 (Good Health and Well-Being) and SDG 11 (Sustainable Cities and Communities), which emphasize the need for resilient urban infrastructure that promotes health and sustainability. Integrating microbial surveillance into public health strategies is essential for achieving these goals, as it enables real-time monitoring of potential risks, such as AMR and the spread of infectious diseases.

Considering the growing emphasis on building smart cities, where technology-driven solutions enhance urban living, we posit that real-time metagenomic analysis can be pivotal in making urban spaces healthier and more resilient. Smart cities are increasingly integrating IoT-based systems for air quality, traffic, and waste management, but microbial surveillance should also be a core component of these ecosystems. Future research should prioritize long-term monitoring of microbial communities in urban environments, enabling city planners to design more adaptive infrastructures that mitigate public health risks while promoting sustainable development.

The unique microbial signatures identified in Chennai’s Metro further emphasize the need for region-specific strategies to address microbial transmission in public spaces. Comparing urban microbiomes across global cities could help policymakers develop targeted cleaning protocols, optimize public health interventions, and manage AMR more effectively. Future research should investigate how human behavior, environmental factors, and urban design impact microbial community composition, and should use this knowledge to develop more resilient, health-conscious urban infrastructure within the broader framework of sustainable and smart city development.

## Data Availability

The raw reads are available in the SRA under accession number PRJNA1201944. The codes used in this article are available in the GitHub repository at https://github.com/IBSE-IITM/MetaSUB-CMRL. The rest of the data supporting this article is provided within the article and its online supplemental material.

## SUPPLEMENTAL MATERIAL

File S1 msphere.00433-26-s0001.docx Extended information about the methods, legends for Fig. S1 and S2, and Tables S1 to S4.

File S2 msphere.00433-26-s0002.xlsx Additional supplemental tables.

Fig. S1 msphere.00433-26-s0003.tiff Bootstrap analysis.

Fig. S2 msphere.00433-26-s0004.tiff Bootstrap analysis controlled for both the number of samples and the sample collection objects.

ASM does not own the copyrights to Supplemental Material that may be linked to, or accessed through, an article. The authors have granted ASM a non-exclusive, world-wide license to publish the Supplemental Material files. Please contact the corresponding author directly for reuse.
