# Supplementary material for: Microbial communities on station and train surfaces in Chennai Metro: insights into urban transit microbiome
Source: mSphere. 2026 Oct 8;11(10):e00433-26. doi: 10.1128/msphere.00433-26 (PMC13711780; doi:10.1128/msphere.00433-26)
Supplement: Supplementary file 1 [file msphere.00433-26-s0001.docx]

### **SUPPLEMENTARY INFORMATION**

## **Microbial communities on station and train surfaces in Chennai Metro: Insights into urban transit microbiome**

Vijaya Yuvaram Singh1,2,3,5,*, Veerendra P. Gadekar2,3,*, Srijith Sasikumar1,2,3, R. M. Rajeeva Lokshanan1,2,3, Senthamizhan V.2, Bharath Prithiviraj4, Himanshu Sinha1,2,3,#, Karthik Raman1,2,3,5,#

1 Department of Biotechnology, Bhupat and Jyoti Mehta School of Biosciences, IIT Madras, Chennai, India

2 Centre for Integrative Biology and Systems Medicine, Wadhwani School of Data Science and Artificial Intelligence, IIT Madras, Chennai, India

3 Wadhwani School of Data Science and Artificial Intelligence, IIT Madras, Chennai, India

4 Reckitt Microbiome & Microbiology R&D Science Platforms, Montvale, New Jersey, USA

5 Department of Data Science and Artificial Intelligence, Wadhwani School of Data Science and Artificial Intelligence, IIT Madras, Chennai, India

*These authors contributed equally to the work.

^#^Corresponding authors: Karthik Raman [kraman@dsai.iitm.ac.in](mailto:kraman@dsai.iitm.ac.in), Himanshu Sinha [sinha@iitm.ac.in](mailto:sinha@iitm.ac.in)

**Supplementary Methods**

1. **Sampling microbial communities in the Chennai Metro stations**

Using the MetaSUB protocol [[1]](https://sciwheel.com/work/citation?ids=16980783&pre=&suf=&sa=0&dbf=0), samples were collected with individually packaged Isohelix Buccal Mini Swabs (MS Mini DNA/RNA Swab, Isohelix, Cat.: MS-02) and stored in barcoded 2D Matrix V-Bottom ScrewTop Tubes (Thermo Scientific, Cat.: 3741-WP1D-BR/1.0mL) pre-filled with 400 µL of Zymo Shield transport and storage solution (Zymo Research, Cat.: R1100) to preserve DNA and RNA. Immediately after collection, swabs were placed in the matrix tubes and stored at -80°C until DNA extraction.

The samples were taken from frequently touched surfaces, including banisters (handrails inside metro stations), kiosks (ticket vending machines), rods (handrails inside trains), and ticket counters (Figure 1B). Four samples were collected per station, totaling 48 per batch. Additionally, one negative control (an air sample) per sample collection batch was collected at the Chennai Airport metro station by holding a dampened Copan swab exposed to the air for approximately 3 minutes, following the MetaSUB consortium protocol [[2]](https://sciwheel.com/work/citation?ids=11114842&pre=&suf=&sa=0&dbf=0). This comprehensive sampling strategy targeted diverse interaction points in the transit environment, providing a robust representation of microbial diversity.

2. **DNA Extraction, Library Preparation, and Sequencing**

DNA extraction was performed using the Microlab STAR Liquid Handling System (Hamilton, Cat.: Microlab STAR) and the ZymoBIOMICS 96 MagBead DNA Kit (Zymo Research, Cat.: D4308) to ensure high-quality recovery of both abundant and low-abundance microbial species. An initial Quality Control (QC) was conducted using the Qubit dsDNA High-Sensitivity Assay. A minimum DNA concentration of 0.9 ng/µL was required for samples to pass QC after extraction. Libraries were prepared using the KAPA HyperPrep Kit (Roche), and sequencing was performed on the Illumina NovaSeq 6000 platform, chosen for its ability to generate high-throughput, paired-end reads suitable for metagenomic analysis. These methods enabled us to maximize the coverage of the microbial diversity present in the samples. Sample processing, sample DNA extraction, and sequencing were performed by MedGenome Labs Ltd., Bangalore, India. Both negative controls did not yield sufficient DNA concentration to pass the QC thresholds and were therefore not sequenced. This suggests minimal detectable environmental or cross-contamination during the sampling and extraction procedures, although the absence of sequenced controls prevented downstream computational decontamination analyses. Out of 96 collected samples, 47 passed QC criteria and were subjected to shotgun metagenomic sequencing for downstream analysis (Figure 1C). Of these 47 samples, six had fewer than 1 million reads after preprocessing and were excluded from further analysis. The remaining 41 samples, with an average of 5.4 million reads, were retained for downstream analyses. A detailed list of the selected 41 samples is provided in Supplementary Table S1.

3. **Preprocessing of the raw reads and mapping**

To characterize the bacterial communities present in Chennai’s metro stations, bacterial taxa were identified through DNA sequencing. The raw Illumina sequencing reads were first assessed for quality using FastQC v0.11.9, and MultiQC v1.13 was employed to aggregate and visualize the quality reports. Preprocessing and assembly of the reads were conducted using modules from the MetaSUB Core Modular Analysis Pipeline (CAMP; https://github.com/MetaSUB-CAMP) [[3]](https://sciwheel.com/work/citation?ids=16981563&pre=&suf=&sa=0&dbf=0). The preprocessing steps included filtering out low-quality bases, low-complexity regions, and short reads using fastp v0.22.0. Adapter sequences were removed from the filtered reads with AdapterRemoval v2.3.3. Error correction was performed on the filtered reads using the Tadpole tool from the BBTools v39.01 aligner package. Tadpole corrected sequencing errors by comparing overlapping reads, identifying mismatches and gaps, and generating a consensus sequence based on the majority of bases. This process reduced noise and improved accuracy by correcting base-calling errors. After preprocessing, the quality of the corrected reads was reassessed using FastQC and MultiQC to ensure the integrity of the dataset. Finally, the processed reads were mapped to host reference genomes, including the human genome assembly (GRCh38) and the mouse genome assembly (MM39), using Bowtie2 v2.4.5 with the ‘very-sensitive’ flag (Table S3). Reads mapped to the host genomes were removed to focus on microbial sequences. After preprocessing, we selected 41 samples for analysis that had >1 million reads.

4. **Taxonomic classification and identification of the core and sub-core species**

For taxonomic classification and determination of species relative abundance across all metagenomic samples, we used Kraken2 v2.1.3 and Bracken v2.7 from the CAMP pipeline. Kraken2 matched individual k-mers within query sequences to the lowest common ancestor (LCA) among all genomes containing the respective k-mer in the kraken2 database v0.1.1 made available by CAMP. To estimate species-level relative abundance, we used Bracken v2.7, which probabilistically redistributed reads from higher taxonomic levels to refine abundance estimates. We defined the core and sub-core species based on the distribution of taxa prevalence across our dataset. Here, prevalence refers to the fraction of samples in which a particular taxon was found at the specific relative abundance threshold. This combined Kraken2-Bracken approach was chosen for its ability to balance computational efficiency with taxonomic depth, a critical feature for analyzing large, diverse datasets like those generated in this study. By leveraging these tools, we ensured both speed and accuracy in taxonomic classification, which was essential for subsequent comparative analyses with global urban microbiome datasets.

5. **Comparative analysis against the global urban microbiome composition**

To compare the microbiome profile of Chennai with those of other major global cities, we utilized data from the global metagenomic map of urban microbiomes provided by the MetaSUB consortium [[2]](https://sciwheel.com/work/citation?ids=11114842&pre=&suf=&sa=0&dbf=0). We performed a batch download of clean read FASTQ files from the MetaSUB central data repository using Geoseeq v0.2.8 [[4]](https://sciwheel.com/work/citation?ids=17676952&pre=&suf=&sa=0&dbf=0). These clean reads were pre-processed through the CAP2 pipeline, which included filtering out low-quality bases and removing host reads. In total, 2,515 samples from 38 cities were initially obtained based on data availability at the time of download. Using the associated metadata for each sample, we excluded those originating from air or biological sources, focusing only on surface samples. This filtering step yielded a final dataset comprising 2,461 samples from 31 cities (Supplementary File 2, Sheet 7). The city with the fewest samples contributed 12, and the total number of samples used per city in our analysis is listed in Table S4. We conducted taxonomic classification analysis on the selected samples using Kraken2 and Bracken as implemented in the CAMP pipeline, consistent with our approach for the Chennai city samples. This allowed us to classify detected species in the Chennai dataset into core, sub-core, peripheral, and unique-core categories across different relative abundance thresholds (Table S2). Subsequently, we compared these species across all cities, including Chennai, to detect any unique signatures specific to Chennai and to identify the sample origin locations. To account for variability in sample numbers across cities and sampling objects, we performed a bootstrap-based resampling analysis. Specifically, we conducted 100 resampling trials in which 41 samples were randomly selected from other cities to match the Chennai sample size. Core and sub-core species were recalculated for each trial and compared against the Chennai-specific unique-core species. Additionally, we focused our sample collection on specific sites, exclusively using samples from kiosks, handrails, escalator handrails, ticket machines, touchscreens, and poles. This filter reduced the MetaSUB samples from 2,461 to 700.

6. **Diversity analysis**

To assess species richness (the variety of species) and evenness (their relative abundance), providing insights into microbial ecosystem differences across environments, we conducted diversity analyses using the phyloseq v1.44.0 [[5]](https://sciwheel.com/work/citation?ids=3722481&pre=&suf=&sa=0&dbf=0) package in R. The phyloseq package integrates with the vegan package for calculating alpha diversity metrics, while ggpubr v0.6.0 [[6]](https://sciwheel.com/work/citation?ids=16999710&pre=&suf=&sa=0&dbf=0) was used for visualization. Alpha Diversity (within-sample diversity) was quantified using the Shannon Index, which measures species distribution entropy. Higher values indicate greater diversity by accounting for both richness and evenness. We used the Wilcoxon test for pairwise comparisons and the Kruskal-Wallis test for group comparisons. The p-values from the pairwise comparisons were adjusted (Padj) using the Benjamini-Hochberg (BH) method in R. Beta Diversity (between-sample diversity) was analyzed using the “ordinate” function in phyloseq, applying Bray-Curtis dissimilarity and Principal Coordinate Analysis (PCoA). Bray-Curtis dissimilarity accounts for taxonomic abundance, providing a sensitive measure for detecting differences between samples. “One-versus-rest” comparison was performed for each of the four sample collection surfaces using the PERMANOVA test.

7. **Differential taxonomic profiles across sample collection objects**

To examine variations in microbial community composition across sample collection objects, we performed a differential abundance analysis using MaAsLin2 v1.15.1 [[7]](https://sciwheel.com/work/citation?ids=12027631&pre=&suf=&sa=0&dbf=0). We employed a “one-versus-rest” approach, comparing each surface type (kiosks, rods, banisters, ticket counters) against all other surfaces combined to identify taxa significantly enriched on each surface. Specifically, for kiosks, we conducted separate comparisons against rods, banisters, and ticket counters. MaAsLin2 uses Centered log ratio (CLR) for normalization and applied a generalized linear model to normalize relative abundance data. To robustly identify surface-specific microbial signatures while accounting for potential spatial and temporal confounding factors, we utilized a mixed-effects modeling approach. Specifically, surface type and sample collection batch were included as fixed effects, while the metro station was included as a random effect to account for spatial clustering. Taxa with an absolute log-fold change |Log_2_FC| > 1 and a Padj < 0.05 were considered significantly differentially abundant. This method enabled us to characterize and quantify microbial differences associated with surface types, highlighting key taxa that are influenced by the collection environment.

8. **Metagenomic assembly and genome reconstruction**

To reconstruct microbial genomes from the metagenomic data, we first assembled the processed sequencing reads using MetaSPAdes v3.15.5 [[8]](https://sciwheel.com/work/citation?ids=3334938&pre=&suf=&sa=0&dbf=0). This produced a collection of contigs and scaffolds representing genomic fragments recovered from the samples. Following the assembly, MetaBAT2 v2.2.7 [[9]](https://sciwheel.com/work/citation?ids=7996543&pre=&suf=&sa=0&dbf=0) was employed to bin metagenome-assembled genomes (MAGs). MetaBAT2 used two main metrics to accurately cluster contigs. It relied on the coverage depth per contig and the tetranucleotide frequency (TNF) patterns. Coverage depth reflected the frequency of nucleotide sequencing, which was determined by aligning raw reads to the assembled contigs. TNF patterns, which represent the frequency of all possible four-nucleotide sequences within a contig, helped distinguish contigs from different microbial genomes based on their genomic composition. To evaluate the quality of the reconstructed MAGs, we used CheckM v1.0.1 [[10]](https://sciwheel.com/work/citation?ids=1028024&pre=&suf=&sa=0&dbf=0). This tool assesses genome completeness and contamination by analyzing lineage-specific marker genes, providing crucial insights into the reliability of the MAGs for downstream biological inferences.

9. **Pangenome analysis**

To explore genetic diversity within the microbial population, we conducted a pangenome analysis that compared multiple genomes to identify core genes shared across genomes and variable genes unique to individual genomes or subsets of genomes. We selected MAGs with completeness greater than 90% and contamination levels below 5%. Taxonomic annotations of the selected MAGs were performed using GTDB-Tk v2.1.1 [[11]](https://sciwheel.com/work/citation?ids=7833838&pre=&suf=&sa=0&dbf=0), and reference genomes for identified organisms were retrieved from NCBI. We used Anvi’o v8 [[12]](https://sciwheel.com/work/citation?ids=917908&pre=&suf=&sa=0&dbf=0), a tool that facilitates visualization, gene and functional annotation, and enrichment analysis across genomes using the Clusters of Orthologous Genes (COG) database (COG2020 [[13]](https://sciwheel.com/work/citation?ids=11165106&pre=&suf=&sa=0&dbf=0)). We additionally constructed a phylogenomic tree using GToTree v1.8.4 [[14]](https://sciwheel.com/work/citation?ids=7067025&pre=&suf=&sa=0&dbf=0). This tree was based on 15 single-copy gene Hidden Markov Models (HMMs) derived from the Pfam database and NCBI, providing insights into the evolutionary relationships among the genomes under study.

10. **Assessing the gene abundance profile of the AMR gene family**

To assess the antibiotic resistance potential of the microbial communities present in the metro stations, we generated the antibiotic resistance profile of the samples using the Resistome Gene Identifier (RGI) tool v6.0.2 from the Comprehensive Antibiotic Resistance Database (CARD) [15] database v3.2.6. RGI-bwt function from the RGI tool aligned the short reads to CARD’s protein homolog models using the k-mer alignment (KMA) read aligner and provided a table of identified drug classes, the annotation for the drug resistance mechanisms, AMR gene family, the number of reads mapped, and the coverage. To mitigate the risk of false positives inherent in short-read mapping, we implemented a stringent filtering criterion on the coverage of the AMR genes. An AMR gene was reported only if the aligned reads covered >80% of the corresponding reference sequence. The read counts from RGI-bwt were normalized by total reads per sample and gene length to calculate RPKM values, and the abundance levels of AMR genes across different antibiotic classes were estimated using CARD. We calculated the prevalence of AMR gene families in our sample set by identifying those present across various drug classes and resistance mechanisms. To reduce potential noise and focus on gene abundance, we put a threshold at the 25th percentile (Q1) of RPKM. For a given drug class or drug resistance mechanism, the prevalence was 100% if at least one AMR gene family was present in all samples. Further, we also used the 2019 WHO AWaRe (Access, Watch, Reserve) classification framework [16] to categorize antibiotic classes associated with the identified AMR genes.

**Supplementary Figures**

Figure S1: The bootstrap analysis shows no significant overlap between the unique core of Chennai and the core and sub-core of any other cities. This analysis is controlled for the number of samples used.

Figure S2: The bootstrap analysis controlled for both the number of samples and the sample-collection objects, showing no significant overlap between the unique-core of Chennai and the core and sub-core of any other cities.

**Supplementary Tables**

Table S1: Number of samples that passed QC, were sequenced, and were used for analysis per metro station

| **S.No** | **Metro station** | **No of samples**  **Dec-2021** | **No of samples**  **Jan-2022** |
| --- | --- | --- | --- |
| 1 | Airport | 0 | 2 |
| 2 | Meenambakkam | 0 | 1 |
| 3 | Alandur | 1 | 2 |
| 4 | Guindy | 0 | 1 |
| 5 | Saidapet | 0 | 2 |
| 6 | Teynampet | 0 | 3 |
| 7 | Central Station | 3 | 4 |
| 8 | Washermanpet | 2 | 4 |
| 9 | Egmore | 2 | 2 |
| 10 | Thirumangalam | 2 | 2 |
| 11 | CMBT | 3 | 3 |
| 12 | Vadapalani | 1 | 1 |
| 13 | Negative control | 0 | 0 |
| 14 | **Total** | **14** | **27** |

Table S2: Number of detected species across different relative abundance thresholds (scale 0-1) within each species category (core, sub-core, peripheral, and unique-core) in the Chennai dataset.

| **Species Category** | **Relative abundance** | | | |
| --- | --- | --- | --- | --- |
|  | **> 0.1** | **> 0.01** | **> 0.001** | **> 0.0001** |
| Core | 0 | 2 | 30 | 495 |
| Sub-core | 0 | 1 | 30 | 481 |
| Peripheral | 5 | 47 | 334 | 823 |
| Unique-core | 0 | 0 | 9 | 16 |

Table S3: Total reads per sample after removing human and mouse reads

| **S.No** | **Sample ID** | **Number of reads** |
| --- | --- | --- |
| 1 | 368257968 | 61,37,371 |
| 2 | 368258054 | 17,94,329 |
| 3 | 368259600 | 23,97,440 |
| 4 | 368259989 | 64,86,810 |
| 5 | 368273379 | 37,30,160 |
| 6 | 368281318 | 28,51,412 |
| 7 | 368281322 | 14,96,154 |
| 8 | 368281337 | 12,25,754 |
| 9 | 368281369 | 54,57,282 |
| 10 | 368281482 | 51,12,232 |
| 11 | 368281490 | 73,14,928 |
| 12 | 368281495 | 63,07,599 |
| 13 | 368281511 | 1,01,32,285 |
| 14 | 368281532 | 25,55,890 |
| 15 | 368246110 | 89,43,782 |
| 16 | 368253732 | 1,15,30,804 |
| 17 | 368258345 | 74,38,425 |
| 18 | 368258351 | 57,23,807 |
| 19 | 368258353 | 67,44,363 |
| 20 | 368258369 | 68,78,396 |
| 21 | 368258383 | 72,75,716 |
| 22 | 368258393 | 66,10,649 |
| 23 | 368258417 | 44,16,381 |
| 24 | 368258423 | 71,63,138 |
| 25 | 368258431 | 77,06,800 |
| 26 | 368259698 | 33,34,353 |
| 27 | 368259701 | 21,56,282 |
| 28 | 368259706 | 75,59,655 |
| 29 | 368259707 | 61,14,749 |
| 30 | 368259710 | 11,06,067 |
| 31 | 368259718 | 16,56,915 |
| 32 | 368259737 | 44,07,434 |
| 33 | 368259760 | 66,75,378 |
| 34 | 368259764 | 13,90,679 |
| 35 | 368259770 | 72,56,608 |
| 36 | 368259776 | 75,24,716 |
| 37 | 368260393 | 63,85,754 |
| 38 | 368297838 | 12,32,670 |
| 39 | 368297861 | 67,20,967 |
| 40 | 368299257 | 99,11,130 |
| 41 | 368299282 | 62,76,148 |

Table S4: Number of samples used per city from MetaSUB

| **S.No.** | **City** | **Number of samples** |
| --- | --- | --- |
| 1 | Hong Kong | 652 |
| 2 | London | 421 |
| 3 | Ilorin | 195 |
| 4 | Singapore | 180 |
| 5 | New York City | 148 |
| 6 | Porto | 111 |
| 7 | Barcelona | 96 |
| 8 | Kyiv | 74 |
| 9 | Doha | 66 |
| 10 | Seoul | 60 |
| 11 | Zurich | 52 |
| 12 | Lisbon | 51 |
| 13 | Stockholm | 43 |
| 14 | Denver | 37 |
| 15 | Berlin | 36 |
| 16 | Offa | 17 |
| 17 | Santiago | 17 |
| 18 | Naples | 16 |
| 19 | Paris | 16 |
| 20 | Sacramento | 16 |
| 21 | Hamilton | 16 |
| 22 | Marseille | 16 |
| 23 | Sofia | 16 |
| 24 | Vienna | 15 |
| 25 | Sendai | 14 |
| 26 | Brisbane | 14 |
| 27 | Kuala Lumpur | 14 |
| 28 | Hanoi | 14 |
| 29 | Auckland | 13 |
| 30 | Minneapolis | 13 |
| 31 | Baltimore | 12 |

**REFERENCES**

[1. Resources - MetaSUB. https://metasub.org/resources/. Accessed 29 Sep 2024.](https://sciwheel.com/work/bibliography/16980783)

[2. Danko D, Bezdan D, Afshin EE, Ahsanuddin S, Bhattacharya C, Butler DJ, et al. A global metagenomic map of urban microbiomes and antimicrobial resistance. Cell. 2021;184:3376-3393.e17.](https://sciwheel.com/work/bibliography/11114842)

[3. Mak L, Tierney B, Wei W, Ronkowski C, Toscan RB, Turhan B, et al. CAMP: A modular metagenomics analysis system for integrated multi-step data exploration. BioRxiv. 2025.](https://sciwheel.com/work/bibliography/16981563)

[4. GeoSeeq - The tools to protect against global public health threats. https://portal.geoseeq.com/. Accessed 25 Mar 2025.](https://sciwheel.com/work/bibliography/17676952)

[5. McMurdie PJ, Holmes S. Phyloseq: a bioconductor package for handling and analysis of high-throughput phylogenetic sequence data. Pac Symp Biocomput. 2012;:235-46.](https://sciwheel.com/work/bibliography/3722481)

[6. ggplot2 Based Publication Ready Plots • ggpubr. https://rpkgs.datanovia.com/ggpubr/. Accessed 6 Oct 2024.](https://sciwheel.com/work/bibliography/16999710)

[7. Mallick H, Rahnavard A, McIver LJ, Ma S, Zhang Y, Nguyen LH, et al. Multivariable association discovery in population-scale meta-omics studies. PLoS Comput Biol. 2021;17:e1009442.](https://sciwheel.com/work/bibliography/12027631)

[8. Nurk S, Meleshko D, Korobeynikov A, Pevzner PA. metaSPAdes: a new versatile metagenomic assembler. Genome Res. 2017;27:824-34.](https://sciwheel.com/work/bibliography/3334938)

[9. Kang DD, Li F, Kirton E, Thomas A, Egan R, An H, et al. MetaBAT 2: an adaptive binning algorithm for robust and efficient genome reconstruction from metagenome assemblies. PeerJ. 2019;7:e7359.](https://sciwheel.com/work/bibliography/7996543)

[10. Parks DH, Imelfort M, Skennerton CT, Hugenholtz P, Tyson GW. CheckM: assessing the quality of microbial genomes recovered from isolates, single cells, and metagenomes. Genome Res. 2015;25:1043-55.](https://sciwheel.com/work/bibliography/1028024)

[11. Chaumeil P-A, Mussig AJ, Hugenholtz P, Parks DH. GTDB-Tk: a toolkit to classify genomes with the Genome Taxonomy Database. Bioinformatics. 2019;36:1925-7.](https://sciwheel.com/work/bibliography/7833838)

[12. Eren AM, Esen ÖC, Quince C, Vineis JH, Morrison HG, Sogin ML, et al. Anvi’o: an advanced analysis and visualization platform for ’omics data. PeerJ. 2015;3:e1319.](https://sciwheel.com/work/bibliography/917908)

[13. Galperin MY, Wolf YI, Makarova KS, Vera Alvarez R, Landsman D, Koonin EV. COG database update: focus on microbial diversity, model organisms, and widespread pathogens. Nucleic Acids Res. 2021;49:D274-81.](https://sciwheel.com/work/bibliography/11165106)

[14. Lee MD. GToTree: a user-friendly workflow for phylogenomics. Bioinformatics. 2019;35:4162-4.](https://sciwheel.com/work/bibliography/7067025)

[15. McArthur AG, Waglechner N, Nizam F, Yan A, Azad MA, Baylay AJ, et al. The comprehensive antibiotic resistance database. Antimicrob Agents Chemother. 2013;57:3348-57.](https://sciwheel.com/work/bibliography/926915)

16. World Health Organization and others, WHO AWaRe Classification Database of Antibiotics for evaluation and monitoring of use. 2019. https://www. who. int/news/item/01-10-2019-who-releases-the-2019-awareclassification-antibiotics
